# Prospective assessment of inter- or intra-fractional variation according to body weight or volume change in patients with head and neck cancer undergoing radiotherapy

**DOI:** 10.1371/journal.pone.0216655

**Published:** 2019-05-16

**Authors:** Seong Hoon Kim, Se An Oh, Ji Woon Yea, Jae Won Park

**Affiliations:** 1 Department of Radiation Oncology, Yeungnam University College of Medicine, Nam-gu, Daegu, South Korea; 2 Department of Radiation Oncology, Yeungnam University Medical Center, Nam-gu, Daegu, South Korea; University of California, San Francisco, UNITED STATES

## Abstract

This study aimed to prospectively investigate the association between body weight (ΔBW) or body volume variations (ΔBV) and inter- or intra-fractional variations (Δ(inter) or Δ(intra)) in patients with head and neck cancer (HNC) undergoing radiotherapy (RT). This study enrolled patients with HNC from December 2015 to December 2017. All patients underwent curative intensity-modulated RT (IMRT) either as definitive or adjuvant treatment. Six-dimensional inter- and intra-fractional variations (Δ(inter) and Δ(intra)) were obtained with ExacTrac (BrainLAB, Feldkirchen, Germany) system. BV was measured 7.5 cm cranio-caudally from the centre using cone beam computed tomography. The BW, BV, and Δ(inter) were calculated based on the value obtained on the first treatment day after each simulation. Both Δ(inter) and Δ(intra) were considered in calculating the optimal margins for planning target volume (PTV), which was calculated using van Herk’s formula. In total, 678 fractions with 39 simulations in 22 patients were analysed. The average ΔBW and ΔBV was -0.43±1.90 kg (range, -7.3 to 5.0) and -24.34±69.0 cc (range, -247.15 to 214.88), respectively. In correlation analysis, Δ(intra) was more associated with ΔBW or ΔBV than Δ(inter). Receiver operating characteristic analysis showed Δ(intra) could differentiate ΔBW from ΔBV, while Δ(inter) could not. The optimal margins for PTV considering both Δ(inter) and Δ(intra) were 3.70 mm, 4.52 mm, and 5.12 mm for the right-left, superior-inferior, and anterior-posterior directions, respectively. In conclusion, the PTV margin of 6 mm for anterior-posterior direction and 5 mm for the other directions were needed. ΔBW or ΔBV correlated with Δ(intra) rather than Δ(inter). Therefore, ΔBW or ΔBV should be assessed for accurate IMRT in patients with HNC.

## Introduction

A multimodal approach that includes radiotherapy (RT) is important for the successful treatment of head and neck cancer (HNC) [1]. Intensity-modulated radiotherapy (IMRT) has recently become the preferred modality for radiotherapy in patients with HNC because it offers more conformal dose distribution. IMRT could spare the parotid glands, thus reducing the incidence of xerostomia [2]. However, the steep dose gradient of an IMRT plan poses a risk of marginal miss due to set-up error [3]. To compensate for this risk, a margin from the clinical target volume (CTV) is added to the planning target volume (PTV). However, although a larger margin lowers marginal misses, it also delivers a higher dose to organs at risk. Therefore, it is necessary to determine the optimal margin required clinically by accurately measuring the error.

Errors that can cause marginal misses can be divided into two types, namely, inter-fractional or intra-fractional variations (Δ(inter) or Δ(intra)). The Δ(inter) is caused by variations of the patient’s body [4] and set-up errors between each daily treatment. To correct for Δ(inter), image-guided radiotherapy (IGRT) is performed, which corrects the patient’s posture pre-treatment. Meanwhile, the Δ(intra) is mainly caused by the fine movement of the patients during RT [5,6].

Although RT is beneficial for HNC, it also results in dysphagia, odynophagia, dry mouth, and a loss of sense of taste, thus often leading to weight loss [7]. Chamchod et al. [8] reported that the mean posttreatment body mass index of patients with cancer is lower than that in pretreatment (28.5 ± 4.9 kg/m^2^ vs 26.2 ± 4.4 kg/m^2^ in men; 27.8 ± 8 kg/m^2^ vs. 26 ± 7.5 kg/m^2^ in women). Furthermore, in cases of cervical lymph node metastasis, the surface of the head and neck can change during RT [9]. Shrinkage of the parotid glands during RT has also been reported [10]. As the surfaces of the head and neck change, a gap may occur between the thermoplastic mask and the skin, which may increase potential error. Lai et al. [11] reported correlations between circumferences at the level of the mastoid tip and inter-fractional variations. However, the measurement in their study was performed only twice in the RT course and was also studied by measuring the length only in two dimensions rather than measuring by body volume (BV) in three-dimensions.

In this study, we measured the variation of body weight (BW, ΔBW), BV (ΔBV), Δ(inter) and Δ(intra) with the aim to determine the relationship between body indices (ΔBW or ΔBV) and set-up errors (Δ(inter) or Δ(intra)) to ultimately suggest an optimal margin for PTV from CTV in RT for HNC.

## Materials and methods

### Eligible patients

This study prospectively enrolled patients with histologically confirmed HNC who underwent curative IMRT either as definitive or adjuvant treatment. The enrolment period started from December 2015 and ended December 2017, and the target number of patients was 25. Those who were aged 18 years of or older were eligible for inclusion. Meanwhile, patients (1) requiring palliative RT, (2) with distant metastases, (3) with malignancies that do not benefit from IMRT (e.g., orbital lymphoma, early glottic cancer), (4) those who had been treated with RT for HNC or who had a history of malignancy other than the HNC for the past 5 years, and (5) those who refused IGRT were excluded.

This study was approved by the Institutional Review Board of Yeungnam University Medical Center (approval number: YUMC 2015-09-034) and was conducted according to the principles expressed in the Declaration of Helsinki. Written informed consent was obtained from all patients.

### Immobilization and simulation

**Fig 1:**
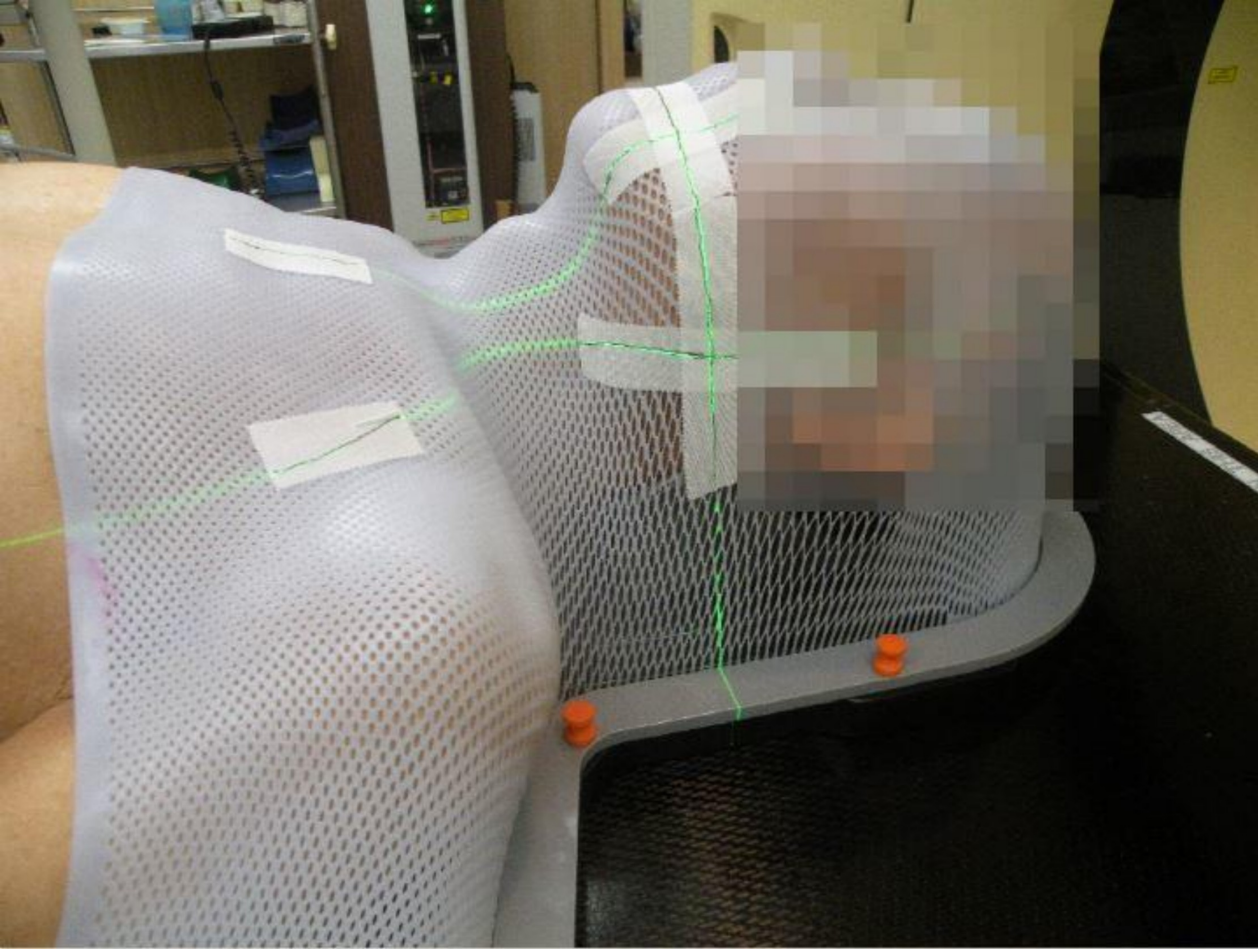
Image of immobilization and set-up. Thermoplast masks were used for immobilization.

DUON^TM^ (Orfit Industries, Wijnegem, Belgium) masks were used as immobilization devices (Fig 1). Bite block was used as needed. All patients were scanned using a Brilliance Big Bore CT simulator (Philips Inc., Cleveland, OH) with a thickness of 2.5 mm. If there was an Δ(intra) error of more than 3 mm in post-treatment ExacTrac (BrainLAB, Feldkirchen, Germany), repeated simulation was performed.

### Treatment planning

An experienced radiation oncologist delineated the gross tumour volume and the CTV for each patient. The PTV was generated with a margin of 5 mm in the CTV. Nine-field static IMRT was generated with inverse planning using anisotropic analytical algorithm. The Eclipse 8.6 (Varian Medical System, Palo Alto, CA, USA) was used as the treatment planning system.

### Image registration and setup protocol

For this study, the kV ExacTrac system and cone beam computed tomography (CBCT) using a kV on-board-imager were used for image guidance. The patients’ BW was measured during each treatment fraction. After the treatment position was set-up, the initial ExacTrac was performed. Image registration was performed using a digitally reconstructed radiograph (DRR) from the CT simulation image as described in our previous study [12]. Image registration was performed automatically based on the bony anatomy of the mandible and cervical spine using the region of interest (ROI) function with spyglass mode, checked by an experienced therapist and then confirmed by an experienced radiation oncologist with an offline review. Then, the correction based on the results of initial ExacTrac was applied to the 6D robotic couch system (BrainLAB, Feldkirchen, Germany). Repositioning was performed as necessary. After correction, pre-treatment ExacTrac and CBCT were performed. Correction via CBCT or pre-treatment ExacTrac was not allowed. Post-treatment ExacTrac was then performed. If an error exceeded 3 mm in any direction of the post-treatment ExacTrac, we performed repeated simulation.

### Treatment delivery

All patients received IMRT treatments using a Novalis Tx (Varian Medical System, CA, USA) linear accelerator machine with HD-120 multi-leaf collimator. Also, photon energy of 6 MV for the radiation dose was delivered to tumour with a dose rate of 600 monitor units per minute.

### Calculation of intra- and inter-fractional errors and body volume measurement

**Fig 2:**
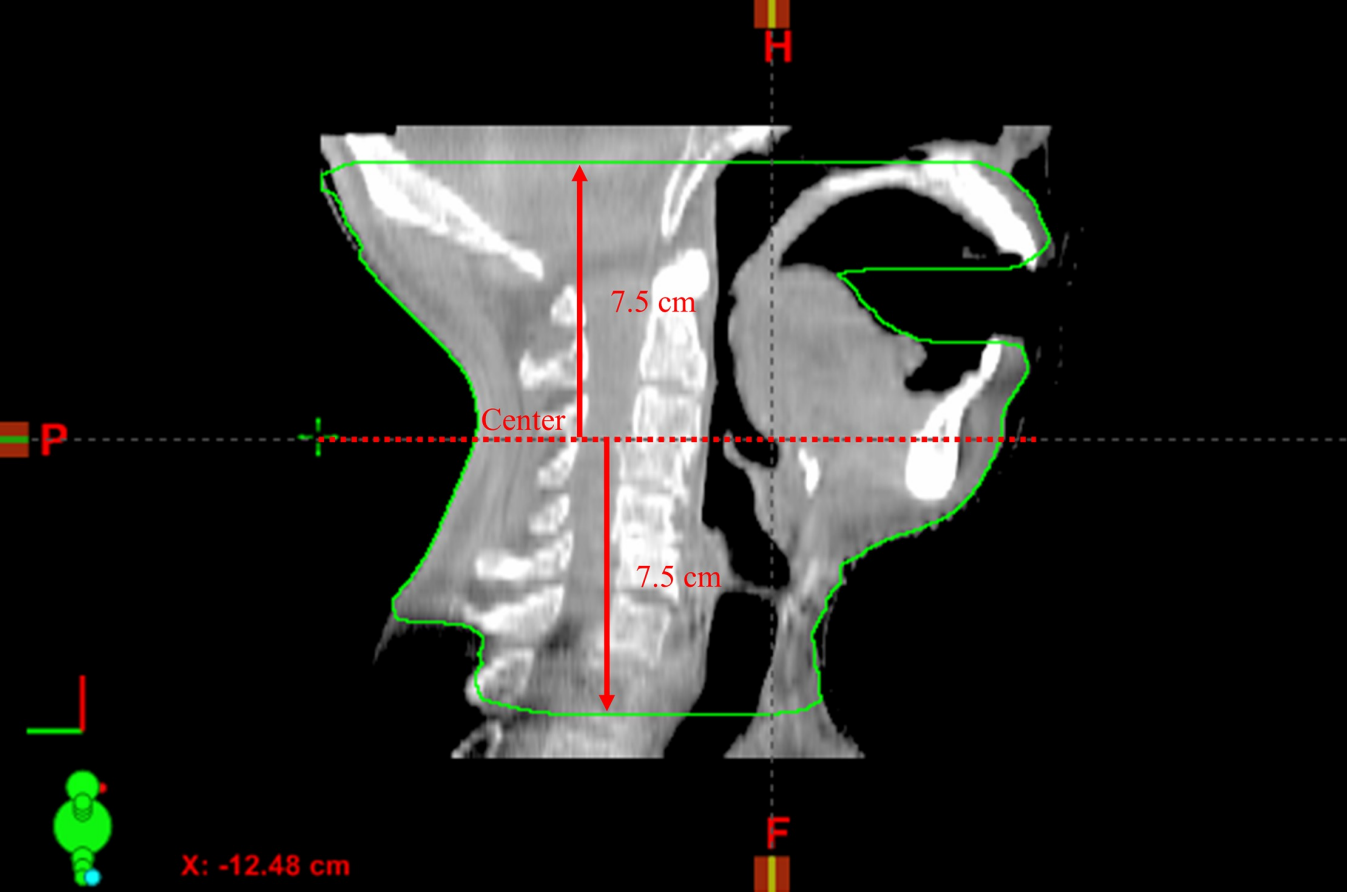
Body volume was measured using cone beam computed tomography. The measurement range was 7.5 cm in the superior and inferior directions from the centre of the CBCT.

**Fig 3:**
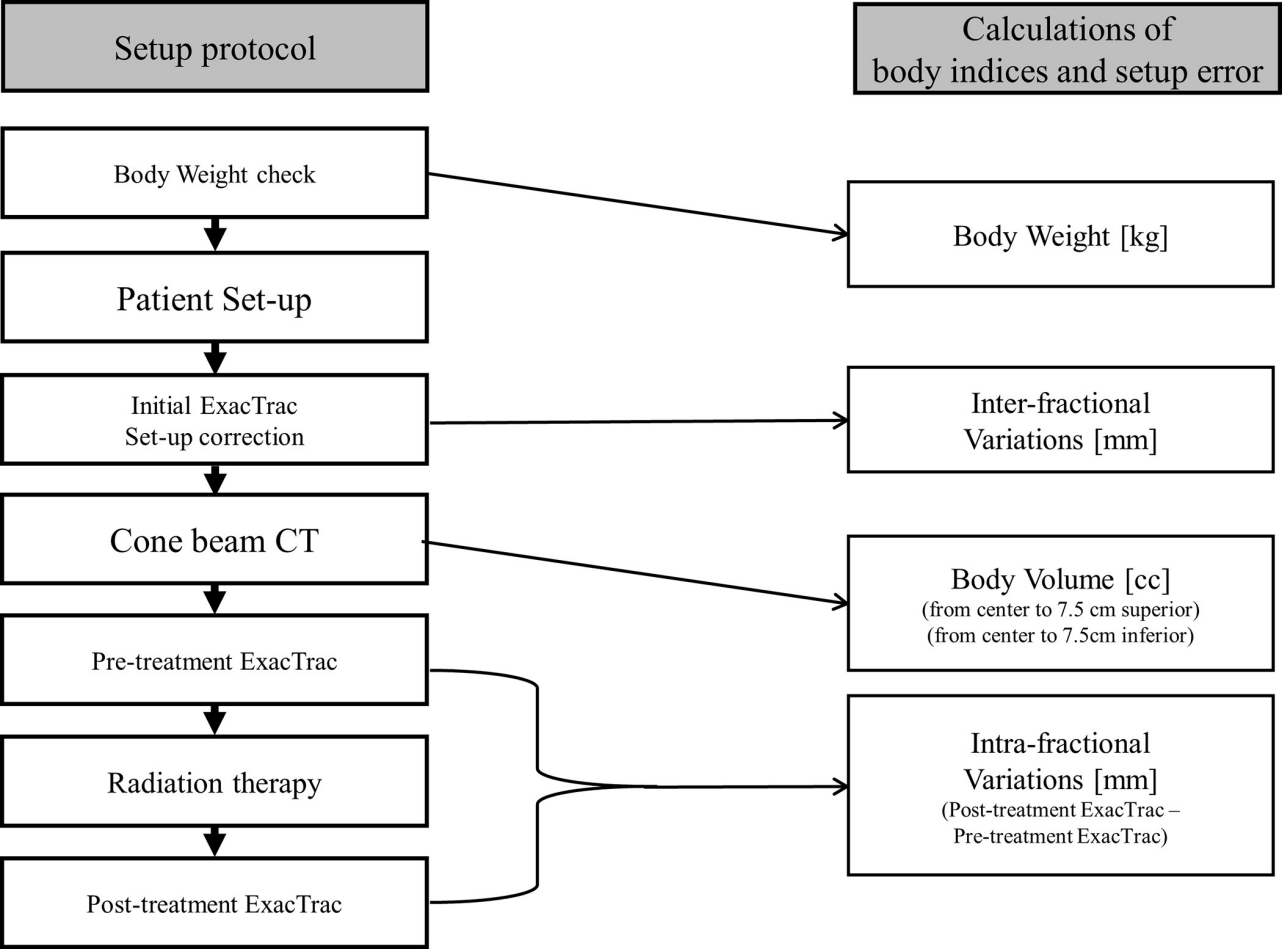
Schema of treatment procedure and calculation of body indices and set-up error.

Six-dimensional (6D; right-left (RL), superior-inferior (SI), anterior-posterior (AP), pitch, roll, and yaw) inter-fractional variations (Δ(inter); ΔRL(inter), ΔSI(inter), ΔAP(inter), Δpitch(inter), Δroll(inter), and Δyaw(inter)) and intra-fractional variations (Δ(intra); ΔRL(intra), ΔSI(intra), ΔAP(intra), Δpitch(intra), Δroll(intra) and Δyaw(intra)) were obtained with the ExacTrac system. The Δ(inter) for RL, SI, AP, pitch, roll, and yaw directions was determined via the initial setup values of ExacTrac, and Δ(intra) for RL, SI, AP, pitch, roll, and yaw directions were obtained by subtracting the ExacTrac setup values of pre-treatment from the ExacTrac setup values of post-treatment. Three-dimensional vector of variations (Δ3D(inter)^2^ = ΔRL(inter)^2^ + ΔSI(inter)^2^ + ΔAP(inter)^2^, Δ3D(intra)^2^ = ΔRL(intra)^2^ + ΔSI(intra)^2^ + ΔAP(intra)^2^) were calculated. Eclipse 8.6 software was used for body volume measurement. The body volume was measured 7.5 cm in the superior and inferior directions from the centre of the CBCT (Fig 2), respectively. The ΔBW and ΔBV were calculated based on the values of body weight and body volume measured by CBCT, which were obtained on the first radiation treatment day. The schema of treatment procedure and calculation of body indices and set-up error is shown in Fig 3.

### Statistics

We used Pearson’s correlation analysis to determine the correlation between ΔBW and ΔBV. We also used Pearson’s correlation analysis for ΔBW or ΔBV and Δ(inter), Δ(intra), absolute value of Δ(inter), and absolute value of Δ(intra). Furthermore, receiver operating characteristic (ROC) analysis was performed to determine the optimal points of ΔBW or ΔBV for treatment in less than 3 mm of Δ(inter) or 2 mm of Δ(intra). Box plot of Δ(inter) or Δ(intra) for each group was created based on ΔBW of 1 kg. All analyses were performed using SPSS version 25.0 (IBM Inc., Armonk, NY, USA).

### Recipes for optimal PTV margins

Margin recipes proposed by Stroom et al. [13] and van Herk et al. [14] were used to calculate the appropriate margin for PTV based on systematic SD (∑) and random SD (σ). Margins were calculated considering both Δ(inter) and Δ(intra). The total ∑ and σ were calculated according to the following equations:

∑^2^ = (∑ of Δ(inter))^2^ + (∑ of Δ(intra))^2^

σ^2^ = (σ of Δ(inter))^2^ + (σ of Δ(intra))^2^

Margin recipes were follows:

Stroom et al.’s [13] formula = 2∑ + 0.7σ

van Herk et al.’s [14] formula = 2.5∑ + 0.7σ

## Results

### Patient characteristics

Twenty-two patients were enrolled. Consequently, 678 fractions with 39 simulations were analysed. The patient characteristics are summarized in Table 1. Two patients (patients numbered 18 and 22) discontinued treatment due to sepsis and patient refusal. During the course of RT, 15 patients lost weight, while 6 patients gained weight. At the end of RT, the average weight changes compared to the start of RT was -2.1±2.7 kg (range, -7.4 to 2 kg).

**Table 1:** Patient characteristics.

No.	Sex	Age	Primary tumour site	BW before RT(kg)	ΔBW during RT (kg)	Total dose (cGy)	Fr.
1	M	76	Tongue	53	0.7	6000	30
2	M	80	Parotid gland	53.9	0.4	6000	30
3	M	63	Oropharynx	62.4	1.6	5040	28
4	M	73	Right retromolar trigone	76.3	-6.4	7000	35
5	M	65	UPMN (Left level IV-V)	46.5	-1.8	6000	30
6	M	53	Floor of Mouth	69.2	-1.7	6000	30
7	M	79	Hypopharynx	59.7	-3.7	7000	35
8	M	64	Glottis	57	-3.6	6600	33
9	M	72	Hypopharynx	59.5	-7.4	7000	35
10	M	61	Subglottis	66	-6	7000	35
11	M	58	Hypopharynx	54.8	-3.8	6600	33
12	M	57	Submandibular gland	53	2	6600	33
13	F	59	Supraglottis	60	-1	6600	33
14	M	69	Tonsil	69.8	0.4	6000	30
15	F	52	Nasopharynx	59	-1	7000	35
16	M	54	Larynx	64	-3	7000	35
17	M	49	Soft palate	72	1	7000	35
18	M	57	Hypopharynx	64	-3	3800	19
19	M	55	Tonsil	61.3	-5.6	7000	35
20	M	73	Supraglottis	66	-2.5	6600	33
21	M	31	Parotid gland	70	-2	6000	30
22	M	66	Supraglottis	44	0	1200	6

Abbreviations: No., registration number; BW, body weight; ΔBW, BW variation; RT, radiotherapy; Fr., fractionation; UPMN, unknown primary metastatic lymph node

### ΔBW and ΔBV

**Fig 4:**
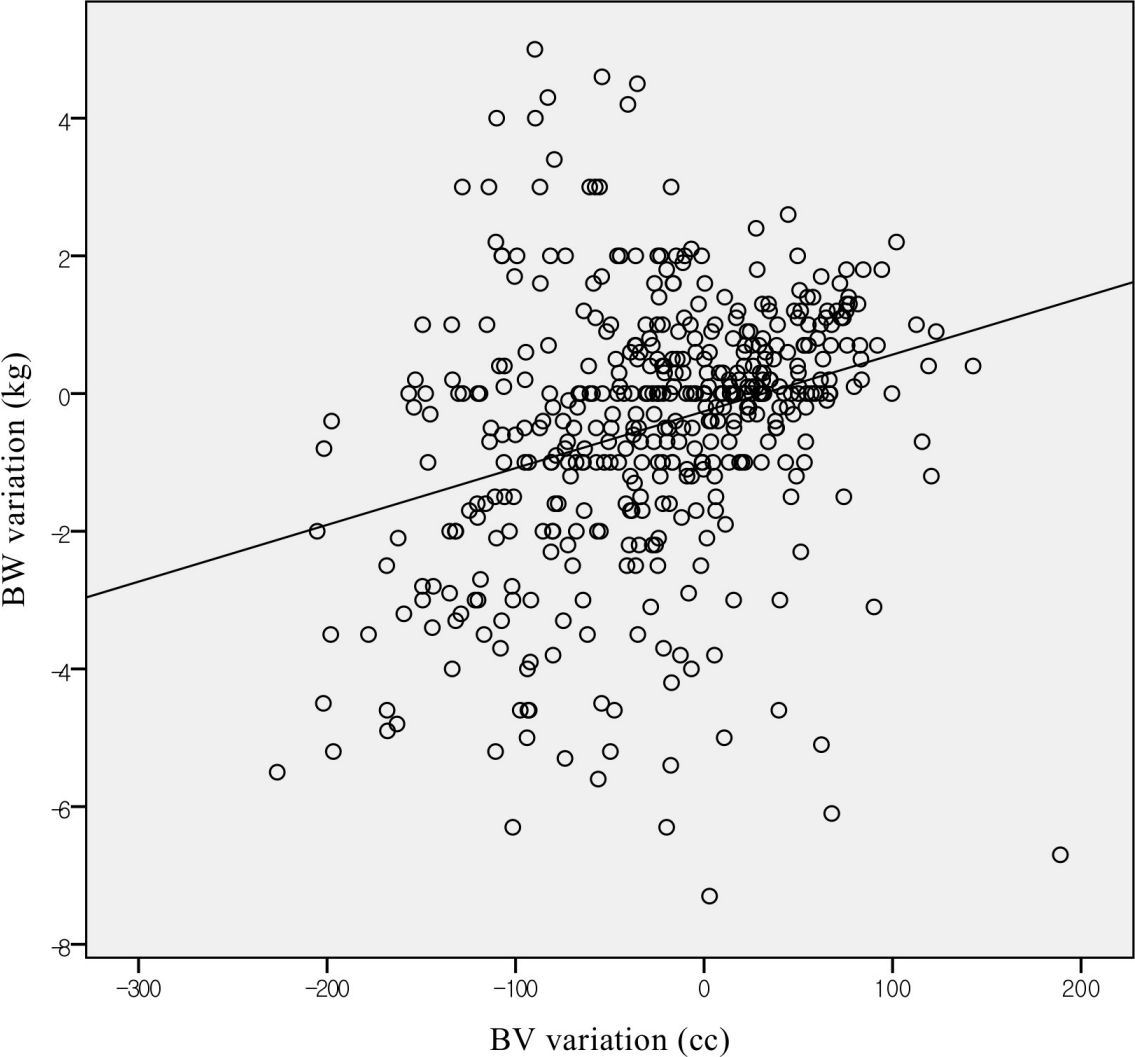
Correlations between body weight variations and body volume variations.

Of the 678 fractions, the ΔBW of 485 fractions (71.5%) and ΔBV of 647 fractions (95.4%) were analysable. The average ΔBW and ΔBV of each fraction was -0.43±1.90 kg (range, -7.3 to 5.0 kg) and -24.34±69.0 cc (range, -247.15 to 214.88 cc), and there was significant correlation between ΔBW and ΔBV (Fig 4, Pearson correlation coefficient (PCC) = 0.291, p<0.001). The BW and BV of each fraction were increased in 183 (37.7% of analysable fractions) and 266 (41.1%) fractions, while they were decreased in 221 (45.6%) and 374 (57.8%) fractions, respectively.

In this study, 6 of the 22 enrolled patients attached with bite block. As the use of bite block may introduce potential bias, we performed additional analyses comparing these patients to those who did not attach with bite block. Because the Kolmogorov-Smirnov test revealed that the data for ΔBV, ΔBW, and Δ (intra) and Δ (inter) were not normally distributed among patients who used bite block (p <0.05), the Mann-Whitney U nonparametric test was used for these variables. When bite block was used, ΔBW was -0.15 ± 1.99 kg; when bite block was not used, ΔBW was -0.6 ± 1.83 kg. Weight loss was greater in patients who did not use bite block (p = 0.001). However, there was no significant difference in ΔBV between patients who used or did not use bite block (-12.2 ± 57.46 cc versus -29.26 ± 72.67 cc, p = 0.072).

### Δ(inter) and Δ(intra)

Descriptive statistics of Δ(inter) and Δ(intra) are shown in Table 2. Among the 678 fractions, Δ(inter) and Δ(intra) were analysable in 677 fractions (99.9%) and 669 fractions (98.7%), respectively. The 3D vectors, which include variations in three directions, were 3.79 ± 2.04 mm (range, 0.28 to 23.80 mm) and 1.14 ± 0.70 mm (range, 0.01 to 5.66 mm) in Δ3D(inter) and Δ3D(intra), respectively.

**Table 2:** Descriptive statistics of inter- and intra-fractional variations.

		Average ± SD	range	Average ±SD	range
				(by absolute value)	
Inter-fractional variation	ΔRL (mm)	0.38 ± 1.73	-7.99 to 12.19	1.32 ± 1.19	0 to 12.19
	ΔSI (mm)	-1.02 ± 2.22	-5.53 to 23.75	1.82 ± 1.63	0 to 23.75
	ΔAP (mm)	-2.19 ± 2.16	-14.4 to 15.85	2.49 ± 1.80	0 to 15.85
	Δpitch (°)	-0.62 ± 0.96	-4.4 to 2.4	0.87 ± 0.74	0 to 4.4
	Δroll (°)	0.03 ± 1.30	-5.6 to 5.8	0.93 ± 0.91	0 to 5.8
	Δyaw (°)	-0.36 ± 1.02	-3.7 to 2.6	0.82 ± 0.71	0 to 3.7
	Δ3D (mm)	3.79 ± 2.04	0.28 to 23.80		
Intra-fractional variation	ΔRL (mm)	0.01 ± 0.76	-2.89 to 2.44	0.56 ± 0.51	0 to 2.89
	ΔSI (mm)	-0.07 ± 0.64	-2.79 to 2.55	0.48 ± 0.42	0 to 2.79
	ΔAP (mm)	-0.02 ± 0.90	-4.74 to 2.99	0.67 ± 0.60	0 to 4.74
	Δpitch (°)	-0.07 ± 0.55	-2.7 to 1.6	0.40 ± 0.40	0 to 2.7
	Δroll (°)	0.05 ± 0.77	-4.2 to 2.9	0.52 ± 0.57	0 to 4.2
	Δyaw (°)	0.03 ± 0.49	-2.1 to 3.0	0.35 ± 0.35	0 to 3.0
	Δ3D (mm)	1.14 ± 0.70	0.01 to 5.66		

Abbreviations: SD, standard deviation; RL, right-left; SI, superior-inferior; AP, anterior-posterior; 3D, three dimensional

### Correlations between Δ(inter) or Δ(intra) and ΔBW or ΔBV

The correlations between ΔBW or ΔBV and Δ(inter) or Δ(intra) are summarized in Tables 3–6. The correlations were significant (p<0.05 and PCC≥0.1 or ≤-0.1) for ΔBW-ΔRL(inter), ΔBW-ΔAP(inter), ΔBW-Δpitch(inter), ΔBV-Δpitch(inter), ΔBW-ΔAP(intra), ΔBW-Δpitch(intra), ΔBW-Δ3D(intra), ΔBV-Δpitch(intra), and ΔBV-Δroll(intra). The PCC between the ΔBW and the ΔAP(intra) was 0.237.

**Table 3:** Correlations between body weight or body volume variations and inter-fractional variations.

		ΔRL(mm)	ΔSI(mm)	ΔAP(mm)	Δpitch(°)	Δroll(°)	Δyaw(°)	Δ3D(mm)
Body weight variations	rho	0.166	-0.027	-0.157	-0.195	-0.019	-0.030	-0.026
	p	0.000	0.547	0.001	0.000	0.677	0.508	0.569
	N	484	484	484	484	484	484	484
Body volume variations	rho	0.018	0.038	-0.041	-0.102	0.011	-0.052	0.062
	p	0.652	0.335	0.304	0.009	0.783	0.190	0.116
	N	646	646	646	646	646	646	646

Abbreviations: ΔRL, variation of right-left; ΔSI, variation of superior-inferior; ΔAP, variation of anterior-posterior; Δpitch, variation of pitch; Δroll, variation of roll; Δyaw, variation of yaw; 3D, three dimensional

**Table 4:** Correlations between body weight or body volume variations and intra-fractional variations.

		ΔRL(mm)	ΔSI(mm)	ΔAP(mm)	Δpitch(°)	Δroll(°)	Δyaw(°)	Δ3D(mm)
Body weight variations	rho	0.020	0.074	0.237	0.164	0.015	0.057	-0.156
	p	0.660	0.105	0.000	0.000	0.743	0.213	0.001
	N	479	0.479	479	479	479	479	479
Body volume variations	rho	-0.043	0.050	0.035	0.135	-0.101	0.106	-0.189
	p	0.284	0.209	0.374	0.001	0.011	0.008	0.000
	N	635	635	635	635	635	635	635

Abbreviations: ΔRL, variation of right-left; ΔSI, variation of superior-inferior; ΔAP, variation of anterior-posterior; Δpitch, variation of pitch; Δroll, variation of roll; Δyaw, variation of yaw; 3D, three dimensional

**Table 5:** Correlations between body weight or body volume variations and absolute value of inter-fractional variations.

Absolute value		ΔRL(mm)	ΔSI(mm)	ΔAP(mm)	Δpitch(°)	Δroll(°)	Δyaw(°)	Δ3D(mm)
Body weight variations	rho	-0.012	-0.111	0.053	-0.072	-0.028	-0.069	-0.026
	p	0.792	0.015	0.248	0.111	0.546	0.131	0.569
	N	484	484	484	484	484	484	484
Body volume variations	rho	-0.006	0.067	0.073	0.089	-0.080	0.078	0.062
	p	0.879	0.088	0.066	0.024	0.041	0.048	0.116
	N	646	646	646	646	646	646	646

Abbreviations: ΔRL, variation of right-left; ΔSI, variation of superior-inferior; ΔAP, variation of anterior-posterior; Δpitch, variation of pitch; Δroll, variation of roll; Δyaw, variation of yaw; 3D, three dimensional

**Table 6:** Correlations between body weight or body volume variations and absolute value of intra-fractional variations.

Absolute value		ΔRL(mm)	ΔSI(mm)	ΔAP(mm)	Δpitch(°)	Δroll(°)	Δyaw(°)	Δ3D(mm)
Body weight variations	rho	-0.110	-0.039	-0.137	-0.027	-0.145	-0.080	-0.156
	p	0.016	0.394	0.003	0.558	0.002	0.079	0.001
	N	479	479	479	479	479	479	479
Body volume variations	rho	-0.108	-0.190	-0.081	-0.146	-0.033	-0.117	-0.189
	p	0.006	0.000	0.040	0.000	0.405	0.003	0.000
	N	635	635	635	635	635	635	635

Abbreviations: ΔRL, variation of right-left; ΔSI, variation of superior-inferior; ΔAP, variation of anterior-posterior; Δpitch, variation of pitch; Δroll, variation of roll; Δyaw, variation of yaw; 3D, three dimensional

Regarding the absolute values of Δ(inter) or Δ(intra), correlations were significant for ΔBW-ΔSI(inter), ΔBW-ΔRL(intra), ΔBW-ΔAP(intra), ΔBW-Δroll(intra), ΔBW-Δ3D(intra), ΔBV-ΔRL(intra), ΔBV-ΔSI(intra), ΔBV-Δpitch(intra), ΔBV-Δyaw(intra), and ΔBV-Δ3D(intra).

### ROC analysis

**Fig 5:**
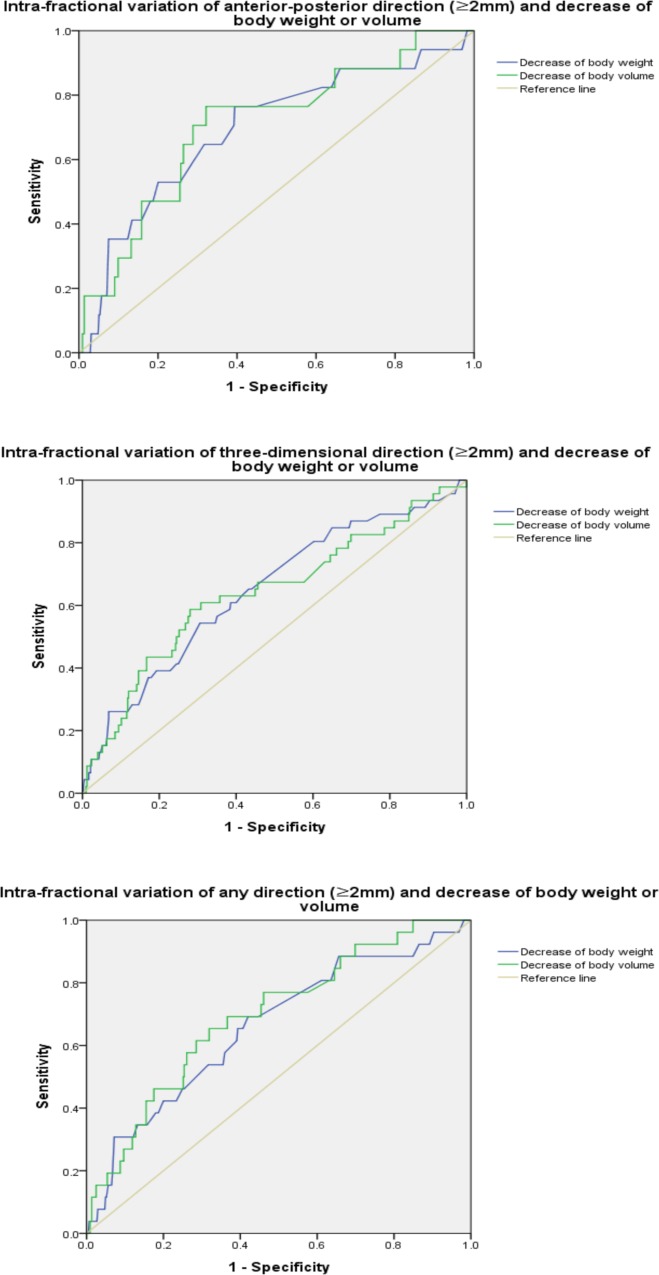
(A) Receiver operating characteristic curve of intra-fractional variations (≥2 mm) of anterior-posterior and decrease of body weight or volume. (B) Receiver operating characteristic curve of intra-fractional variations (≥2 mm) of three-dimensional vector and decrease of body weight or volume. (C) Receiver operating characteristic curve of intra-fractional variations (≥2 mm) of any directions and decrease of body weight or volume.

ROC analysis based on Δ(inter) ≥3 mm and Δ(intra) ≥2 mm of RL, AP, SI, 3D vector, and any directions (when any one of the RL, SI, or AP was outside the specified tolerance) was performed (Table 7). Significance was observed at ΔAP(intra)-decrease of ΔBW (area under the curve (AUC) = 0.694), Δ3D(intra)-decrease of ΔBW (AUC = 0.646), any direction of Δ(intra)-decrease of ΔBV (AUC = 0.707), Δ3D(intra)-decrease of ΔBV (AUC = 0.639), and any direction of Δ(intra)-decrease of ΔBV (AUC = 0.694) (Fig 5A–5C).

**Table 7:** Results of receiver operating characteristic analysis between decrease of body weight or volume and inter- (≥3 mm) or intra-fractional (≥ 2 mm) variations.

				Asymptotic 95% CI	
		AUC	Asymptotic significance	Lower bound	Upper bound
Δ(inter)—decrease of ΔBW	RL	0.558	0.228	0.468	0.649
	SI	0.513	0.670	0.449	0.578
	AP	0.442	0.042	0.381	0.503
	3D	0.469	0.252	0.417	0.520
	Any direction	0.491	0.737	0.439	0.544
Δ(inter)—decrease of ΔBV	RL	0.541	0.400	0.437	0.645
	SI	0.429	0.021	0.366	0.492
	AP	0.425	0.009	0.369	0.482
	3D	0.452	0.078	0.399	0.505
	Any direction	0.435	0.015	0.384	0.487
Δ(intra)—decrease of ΔBW	RL	0.668	0.069	0.490	0.846
	SI	0.459	0.886	0.358	0.559
	AP	0.694	0.007	0.557	0.831
	3D	0.646	0.001	0.559	0.733
	Any direction	0.659	0.006	0.548	0.771
Δ(intra)—decrease of ΔBV	RL	0.663	0.077	0.517	0.810
	SI	0.970	0.104	0.955	0.986
	AP	0.707	0.004	0.579	0.835
	3D	0.639	0.002	0.547	0.731
	Any direction	0.694	0.001	0.592	0.796

Abbreviations: AUC, area under receiver operating characteristic curve; CI, confidence interval; RL, right-left; SI, superior-inferior; AP, anterior-posterior; 3D, three dimensional; Δ(inter), inter-fractional variation; Δ(intra), intra-fractional variation; ΔBW, variation of body weight; ΔBW, variation of body volume

### Comparison of 1 kg group of ΔBW

**Fig 6:**
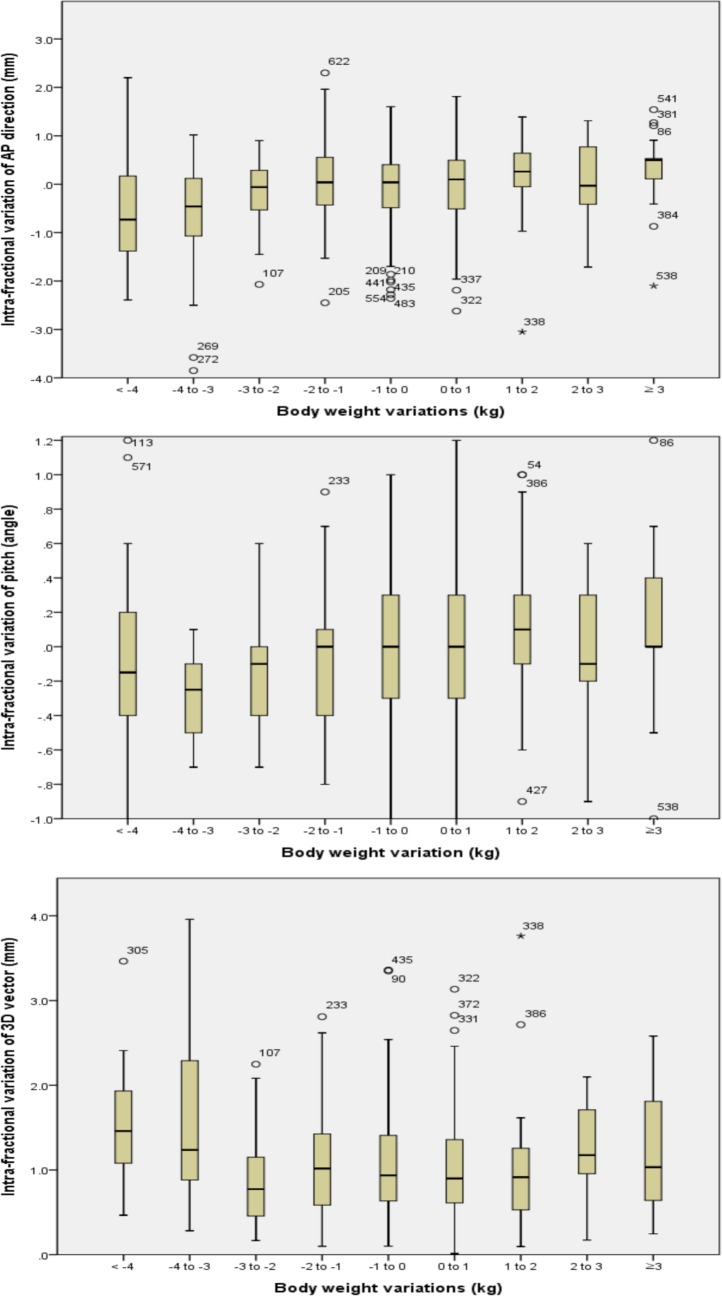
(A) Box plot of intra-fractional variation of anterior-posterior direction per body weight variation group of 1 kg. (B) Box plot of intra-fractional variation of pitch per body weight variation group of 1 kg. (C) Box plot of intra-fractional variation of three-dimensional vector per body weight variation group of 1 kg.

A box plot of the ΔAP(intra), Δ3D(intra), and Δpitch(intra) grouping in groups of 1 kg of ΔBW are shown in Fig 6A–6C. These graphs showed larger variations in patients with weight loss of more than 3 kg. No profound differences were noted in other directions of Δ(intra) and all directions of Δ(inter).

### Optimal margins for the PTV from the CTV

Table 8 summarizes the optimal margins for the PTV based on systematic SD (∑) and random SD (σ) as previously proposed [13,14]. Based on Stroom’s formula, the calculated margins considering Δ(inter) ranged from 2.87 mm to 3.99 mm, and the margins considering both Δ(inter) and Δ(intra) ranged from 3.04 mm to 4.19 mm. Based on van Herk’s formula, the calculated margins are 3.5–4.88 mm and 3.7–5.12 mm, respectively.

**Table 8:** Optimal margins for PTV considering both inter- and intra-fractional variations.

Recipe	Margins considering intra-fractional variations only			Margins considering both inter- and intra-fractional variations		
	RL (mm)	SI (mm)	AP (mm)	RL (mm)	SI (mm)	AP (mm)
2∑ + 0.7σ [13]	2.87	3.66	3.99	3.04	3.75	4.19
2∑ + 0.7σ [14]	3.5	4.41	4.88	3.7	4.52	5.12

Abbreviations: RL, right-left; SI, superior-inferior; AP, anterior-posterior; ∑, standard deviation of systematic errors; σ, standard deviation of random errors

### Repeated simulation due to the post-treatment ExacTrac exceeding 3 mm

**Fig 7:**
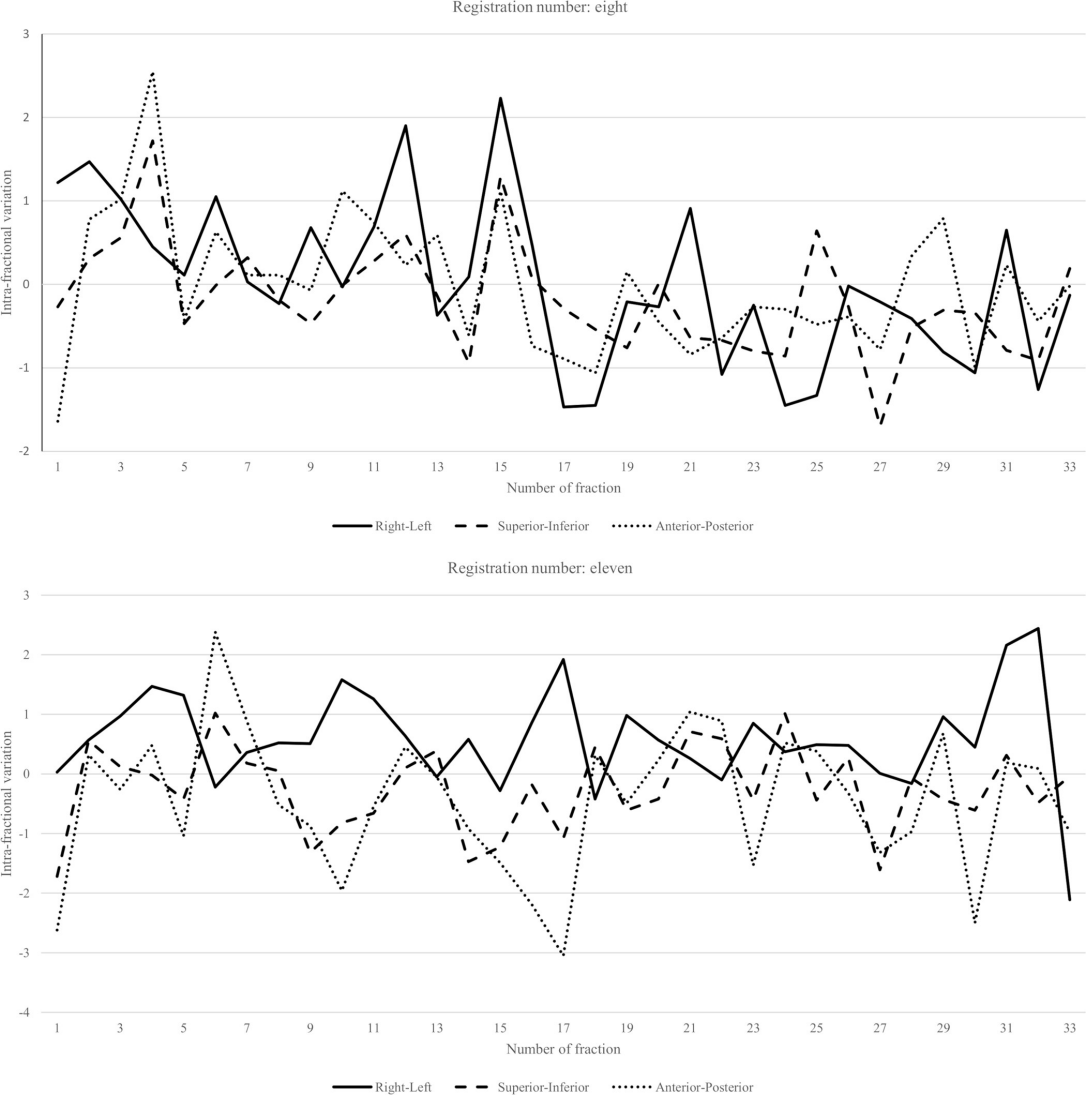
(A) Intra-fractional error of patient number eight. Simulation had to be repeated in this patient after the 15^th^ fraction. The average and standard deviation of three-dimensional intra-fractional variations before and after repeated simulation were 1.37±0.84 mm and 1.19±0.51 mm, respectively. (B) Intra-fractional error of patient number 11. Simulation had to be repeated in this patient after the 17^th^ fraction. The average and standard deviation of three-dimensional intra-fractional variations before and after repeated simulation were 1.73 ± 1.02 mm and 1.46 ± 0.68 mm, respectively.

Repeated simulation was performed when the post-treatment ExacTrac exceeded 3 mm in RL, SI, or AP. Patient number of 8 and 11 required repeated simulations after the 15^th^ and 17^th^ fractions, respectively (Fig 7A and 7B). For both of these patients, there was no planned repeat simulation.

## Discussion

### Optimal margins for the PTV from the CTV

In this study, the ExacTrac system and CBCT were used to calculate the optimal margins from the CTV to the PTV. However, Qi et al.[15] have reported that daily random setup errors and the CTV-to-PTV margins for treatment of head-and-neck cancer were highly affected by IGRT modalities and image quality. To identify the differences between image systems, in our previous study [16], we reported that the clinical setup discrepancy between the ExacTrac system and the CBCT for the intracranial stereotactic radiosurgery was <1.01 mm and <0.82° for online matching.

Assessment of intra-fractional motion with pre- and post-kV imaging as a surrogate of intra-fractional motion is an important issue. However, many studies have previously used multi-modalities such as computed tomography (CT), CBCT, magnetic resonance imaging (MRI), and kV images as surrogates of intra- or intra-fractional motion [4–12]. These studies have assumed that the evaluation of motion trajectory at specific time points, such as before and after treatment, represents inter-or intra setup variation in radiotherapy. Chan et al. [17] has pointed out that continuous fluoroscopic imaging can trace the tumour motion trajectory throughout the radiotherapy treatment, but the patient may receive additional undesired radiation dose. Therefore, we assumed that the kV images before and after treatment sufficiently represented intra-fractional variations.

The criteria for optimal margins in PTV have not been established. In general, the Radiation Therapy Oncology Group protocols recommend a minimum of 5 mm margin around the CTV in all dimensions for HNC patients who did not undergo IGRT [18]. The calculated margins of our study considering only Δ(inter) for the PTV based on Stroom’s and van Herk’s formula [13,14] are generally consistent with those of previous studies. In previous studies, the optimal margins calculated based on van Herk’s formula were 3.4–3.8 mm, 3.8–4.8 mm, and 3.7–4.4 mm in the RL, SI, and AP directions, respectively [19–21] (Table 9). Considering Δ(inter) only, a 5 mm margin for PTV seems to be sufficient in all dimensions.

**Table 9:** Comparisons of calculated margins for planning target volumes with those of previous studies considering inter-fractional variations.

Author	Image guide method	Margin recipe	Calculated margin (mm)		
			RL	SI	AP
Dionisi et al. [19]	CBCT	2.5∑ + 0.7σ	3.48	4.08	4.33
Dzierma et al. [20]	CBCT	2∑ + 0.7σ	3.3	3.3	3.5
		2.5∑ + 0.7σ	3.8	3.8	4.0
Gupta et al. [21]	EPID	2∑ + 0.7σ	3.34	4.14	3.28
		2.5∑ + 0.7σ	3.76	4.74	3.76
This study	ExacTrac	2∑ + 0.7σ	2.87	3.66	3.99
		2.5∑ + 0.7σ	3.50	4.41	4.88

Abbreviation: RL, right-left; SI, superior-inferior; AP, anterior-posterior; CBCT, cone beam computed tomography; EPID, electronic portal imaging device

The calculated margins based on van Herk’s formula considering both Δ(inter) and Δ(intra) were analogous to the results obtained by Cacicedo et al. [22] in the RL (3.7–4.2 mm) and SI (3.9–4.5 mm) directions, but there was a difference of approximately 1 mm in the AP direction (4.1 mm in the study of Cacicedo et al. [22] vs 5.12 mm in our study, Table 10). In our study, ExacTrac was used, while a previous study [22] measured the error using only the AP and lateral field via an electronic portal imaging device. ExacTrac provides 6D information on the error, which is calibrated using the 6D couch, allowing for more accurate measurements of Δ(inter) and Δ(intra).

**Table 10:** Comparisons of calculated margins for planning target volumes with those of previous studies considering both inter- and intra-fractional variations.

Author	Image guide method	Margin recipe	Calculated margin (mm)		
			RL	SI	AP
Cacicedo et al.[22]	EPID	2∑ + 0.7σ	3.6	3.3	3.5
		2.5∑ + 0.7σ	4.2	3.9	4.1
This study	ExacTrac	2∑ + 0.7σ	3.04	3.75	4.19
		2.5∑ + 0.7σ	3.70	4.52	5.12

Abbreviation: RL, right-left; SI, superior-inferior; AP, anterior-posterior; EPID, electronic portal imaging device

### Impact of ΔBW or ΔBV and set-up error (Δ(inter) or Δ(intra))

The correlations between ΔBW and Δ(inter) were significant in several studies [23,24], although some studies reported contrasting results [25,26]. Hou et al. [23] reported that patients with nasopharyngeal cancer with weight loss of >5% had significantly larger ΔAP(inter). Zia et al. [24] found that weight loss was associated with ΔLR(inter). In our study, ΔRL(inter), ΔAP(inter), and Δpitch(inter) were correlated with ΔBW, and Δpitch(inter) was correlated with ΔBV (PCC>0.1 or PCC<-0.1). To ignore the offset due to the direction of error, we analysed the absolute value of Δ(inter). Then, ΔSI(inter) was correlated with absolute value of ΔBW (PCC = -0.111), but no Δ(inter) was correlated with the absolute value of ΔBV.

It is important that Δ(intra) is not neglected. Cacicedo et al. [22] reported that systemic and random Δ(intra) were 0.65–1.11 mm and 1.13–1.16 mm at 3 mm action level, while systemic and random Δ(inter) were 0.77–1.42 mm and 1–1.31 mm, respectively. Gurney-Champion et al. [5] assessed Δ(intra) based on magnetic resonance images, and systemic and random Δ(intra) were <1.4 mm and <2.1 mm 95% of the time. In our study, the average Δ3D was 1.14 mm ± 0.70 (range, 0.01 to 5.66 mm). However, to the best of our knowledge, no study has analysed the relationship between ΔBW or ΔBV and Δ(intra). In our study, ΔAP(intra), Δpitch(intra), and Δ3D(intra) were weakly correlated with ΔBW. Further, Δpitch(intra), Δroll(intra), Δyaw(intra), and Δ3D(intra) were weakly correlated with ΔBV (PCC>0.1 or PCC<-0.1). When compared with the absolute value, ΔRL(intra), ΔAP(intra), Δroll(intra), and Δ3D(intra) were weakly correlated with ΔBW. In addition, ΔRL(intra), ΔSI(intra), Δpitch(intra), Δyaw(intra), and Δ3D(intra) were also correlated with ΔBV. In general, Δ(intra) was more strongly associated with ΔBW or ΔBV than Δ(inter). ROC analysis showed similar results, that is, Δ(intra) was correlated with ΔBW or ΔBV, while Δ(inter) was not. These results indicate the need for further studies on the relationship between ΔBW or ΔBV and Δ(intra) for accurate RT for patients with HNC.

In addition, a box plot of ΔAP(intra) grouping in groups of 1 kg suggests that weight loss >3 kg may result in larger Δ(intra). This result suggests that repeated simulation may be needed when the weight loss is over 3 kg. Because Δ(intra) is an important issue in the RT for HNC patients, further studies should be considered. Recently, Navran et al.[27] reported that reducing the CTV-PTV margin from 5 mm to 3 mm, combined with daily CBCT-guided VMAT, reduced the radiation-related toxicity. Thus, in order to reduce the margin, it is important to maintain body weight during radiotherapy.

The absorbed dose of the image guidance system by multi modalities should be confirmed in image registration and setup protocol. Because single verification with X-ray tube 1 and 2 with ExacTrac system is 1 mSv [12], the overall absorbed dose for the ExacTrac system is 4 mSv with 2mSv for inter-fractional variation, and 2mSv for intra-fractional variations. This dose is lower than the dose of CBCT (14 mSv).

### Study limitations

First, BV measurement using CBCT has some uncertainties. Compared to conventional CT, distinct artefacts present in CBCT could impact BV measurement, although we believe this risk disappears due to the large number of fractions. Second, because BW measurement and CBCT were not performed on all patients, only 71.5% of ΔBW and 95.4% of ΔBV were analysable. In addition, pre-treatment ExacTrac was not performed in one fraction because of a mechanical problem. Moreover, post-treatment ExacTrac was missed in 8 of the 678 fractions. Third, weight loss was smaller than expected. The average ΔBW was -0.43±1.90 kg in all fractions, and ΔBW was increased in 37.7% of the analysable fractions. Also, we performed repeated simulation when the post-treatment ExacTrac showed over 3 mm of variation which reflect Δ(intra). Thus, excessive Δ(intra) was suppressed. As such, it was difficult to observe any significant set-up error.

## Conclusions

The PTV margins of 6 mm for AP direction and 5 mm for the other directions were needed to include 95% of both inter- and intra-fractional set-up variations. Further, BW and BV were correlated with set-up variations. Specifically, intra-fractional variations were positively correlated with changes in BW or BV. Therefore, assessments of BW and BV should be considered for accurate IMRT in patients with HNC.
